# Structural and functional insights into lactobacin A: a novel non-pediocin-like bacteriocin from a *Liquorilactobacillus* strain related to *L. mali*

**DOI:** 10.1128/spectrum.01382-25

**Published:** 2026-03-09

**Authors:** Yumi Komori, Naoya Ozawa, Hiroshi Kuwahara, Mikio Aoki

**Affiliations:** 1 Bioscience Research Laboratory, Sumitomo Chemical Co., Ltd., Osaka, Japan; Peking University People's Hospital, Beijing, China

**Keywords:** lactic acid bacteria, *Lactobacillus*, bacteriocins, antimicrobial peptides

## Abstract

We identified and characterized a novel Class IId bacteriocin, lactobacin A, produced by *Liquorilactobacillus* sp. SC-2001, which was isolated from *Quercus serrata*. The culture supernatant of this strain exhibited a narrow spectrum of antibacterial activity against gram-positive bacteria, including *Streptococcus uberis* and *Latilactobacillus sakei*. Genomic analysis and sugar fermentation pattern revealed that *Liquorilactobacillus* sp. SC-2001 is closely related to *Liquorilactobacillus mali*, making this the first report of a bacteriocin-producing strain within this lineage. The structure of lactobacin A was elucidated through a three-step purification process of the peptide from *Liquorilactobacillus* sp. SC-2001 culture supernatant, followed by N-terminal peptide sequencing and nano liquid chromatography-tandem mass spectrometry (nanoLC/MS/MS)-based *de novo* sequencing and confirmation via RNA sequencing of the coding gene. Sequence analysis demonstrated that lactobacin A is a novel, non-pediocin-like, single-peptide bacteriocin with low similarity to previously reported bacteriocins. Adjacent genes encoding ATP-binding cassette (ABC) transporters were identified, suggesting potential roles in leader peptide cleavage, transport, and self-immunity mechanisms. Antibacterial assays using synthetic peptides revealed that the N-terminal sequence is critical for activity. Notably, lactobacin A exhibited exceptional stability across a wide range of pH levels, elevated temperatures, and oxidative environments. These findings highlight lactobacin A as a promising candidate for the development of novel antimicrobial agents.

**IMPORTANCE:**

Lactobacin A represents a unique addition to the Class IId bacteriocins, characterized by its non-pediocin-like structure and novel functional properties. This study not only introduces the first bacteriocin-producing strain closely related to *Liquorilactobacillus mali* but also underscores the potential of lactobacin A as a stable and effective antimicrobial agent under diverse environmental conditions. Its robust stability and narrow-spectrum activity against gram-positive bacteria make it a valuable candidate for applications in food preservation and therapeutic development.

## INTRODUCTION

Antimicrobial peptides are produced by various organisms and play a critical role in biological defense mechanisms (1, 2). Unlike traditional antibiotics, these peptides act rapidly and degrade into amino acids, reducing their environmental persistence and the risk of resistance development (3, 4). With the growing threat of drug-resistant bacteria, antimicrobial peptides are gaining attention as viable alternatives.

Ribosomally synthesized and post-translationally modified peptides (RiPPs) represent a diverse and rapidly expanding superfamily of natural products with significant biological activities and structural diversity (5, 6). Among RiPPs, bacteriocins are a well-studied group of antimicrobial peptides produced by bacteria, especially lactic acid bacteria (LAB), and have attracted attention for their potential applications in food preservation and medicine (5).

Bacteriocins produced by LAB are classified into three main classes based on their biosynthesis and biological activity (7): Class I (ribosomally produced and post-translationally modified peptides), Class II (unmodified bacteriocins), and Class III (large, heat-labile bacteriocins). Class II bacteriocins are further subdivided into four subclasses: IIa (pediocin-like bacteriocins characterized by strong anti-Listeria activity and a conserved N-terminal motif), IIb (two-peptide bacteriocins composed of two distinct peptides that act synergistically), IIc (leaderless bacteriocins that lack an N-terminal leader sequence and are active immediately after synthesis) (8), and IId (non-pediocin-like single-peptide bacteriocins with diverse structures and modes of action) (6, 7, 9). These subclasses reflect differences in structure, biosynthesis, and antimicrobial mechanisms, underscoring the diversity of Class II bacteriocins.

Nisin A, one of the best-studied Class I bacteriocins, was first reported in 1928 as an antimicrobial substance produced by lactic acid bacteria (10). Its structure, containing meso-lanthionine and 3-methyllanthionine residues, was elucidated in 1971 (11). The ribosomal origin of nisin and related lantibiotics was experimentally verified in 1988 through the sequencing of the biosynthetic gene cluster for epidermin (12, 13). Nisin A is widely used as a food preservative due to its strong activity against gram-positive bacteria. However, its limited stability in neutral to alkaline environments and high temperatures restricts its applications (14, 15). In contrast, bacteriocins with enhanced stability could offer broader utility.

*Liquorilactobacillus mali* is commonly isolated from plant-based fermented foods, such as cider and kefir (16, 17), and has been studied for its health-promoting properties, including anti-obesity and antioxidant effects (18, 19). Despite the identification of bacteriocins in other *Liquorilactobacillus* species, no bacteriocin production has been reported in *L. mali* or its closely related strains (20).

Therefore, this study focuses on the identification and characterization of lactobacin A, a novel bacteriocin produced by *Liquorilactobacillus* sp. SC-2001, a strain closely related to *L. mali*. Our findings highlight the potential of *Liquorilactobacillus* as a source of useful bacteriocins and contribute to the development of new antimicrobial agents for food and therapeutic applications.

## RESULTS AND DISCUSSION

### Identification of *Liquorilactobacillus* sp. SC-2001

A LAB strain was isolated from *Quercus serrata* and identified as motile, gram-positive, rod-shaped, and negative for catalase and oxidase reactions. The 16S rRNA gene sequence showed 99% identity with *Liquorilactobacillus mali* NBRC102159 (21)*,* 98% with *Liquorilactobacillus cacaonum* LMG24285, and 97% with *Liquorilactobacillus aquatius* IMCC1736. Sugar fermentation tests using the API kit revealed similarities to *L. mali*, with slight differences in mannitol fermentation (Table S1). The strain grew at 15°C and did not exhibit arginine dihydrolase activity (Table S2). These findings suggest that *Liquorilactobacillus* sp. SC-2001 is closely related to *L. mali* but represents a distinct strain.

The culture supernatant of *Liquorilactobacillus* sp. SC-2001 exhibited antibacterial activity against several gram-positive strains, including *Streptococcus uberis*, highlighting its potential as a source of novel antimicrobial compounds (Table 1).

**TABLE 1:** Antimicrobial activity of *Liquorilactobacillus* sp. SC-2001

Strain	Medium^*a*^	Activity^*b*^
*Staphylococcus aureus* ATCC 6538	CAMHB	+
*Staphylococcus pseudintermedius* JCM 17571	CAMHB	+
*Streptococcus uberis* JCM 5709	CAMHB with lysed horse blood (2.5%)	++
*Bacillus cereus* ATCC 14579	CAMHB	−
*Bacillus subtilis* NBRC 13719	CAMHB	+
*Bacillus coagulans* NBRC 12583	CAMHB	−
*Bacillus licheniformis* NBRC 12200	CAMHB	+
*Lactiplantibacillus plantarum* NBRC 101973	MRS	−
*Listeria monocytogenes* ATCC 15313	CAMHB with lysed horse blood (2.5%)	−
*Escherichia coli* ATCC 25922	CAMHB	−

^*a*^ CAMHB, cation-adjusted Mueller-Hinton broth; MRS, de Man–Rogosa–Sharpe agar; GAM, Gifu anaerobic medium; Supplemented Brucella broth, Brucella broth + 5 μg/mL hemin + 1 μg/mL vitamin K1 + 5% lysed horse blood.

^*b*^ −, no antibacterial activity (MIC > 1/10 dilution of supernatant); +, antibacterial activity (MIC = 1/10 dilution); ++, strong antibacterial activity (MIC = 1/20 dilution).

### Purification and structural analysis of bacteriocin from *Liquorilactobacillus* sp. SC-2001

**Fig 1:**
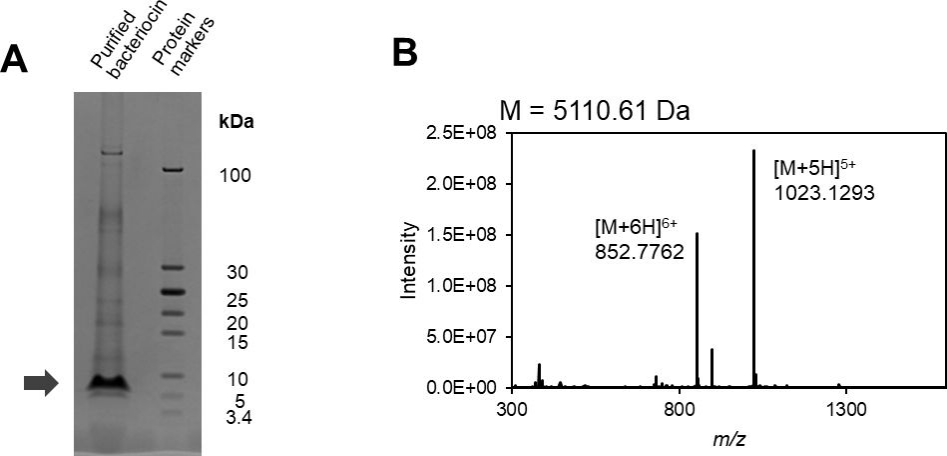
Analysis of purified bacteriocin from *Liquorilactobacillus* sp. SC-2001. (**A**) SDS-PAGE of protein markers compared to purified bacteriocin with molecular weights between 3.4 and 100 kDa. (**B**) Mass spectrum of purified bacteriocin. Multiple charged molecular ions are indicated.

Lactobacin A was purified through a three-step process involving ammonium sulfate precipitation, cation exchange chromatography, and reverse-phase chromatography. Sodium dodecyl sulfate polyacrylamide gel electrophoresis (SDS-PAGE) analysis revealed a single diffuse band corresponding to a molecular weight of approximately 5–10 kDa (Fig. 1A). Liquid chromatography-mass spectroscopy (LC/MS) analysis confirmed the molecular weight as 5,110.61 Da (Fig. 1B).

**Fig 2:**
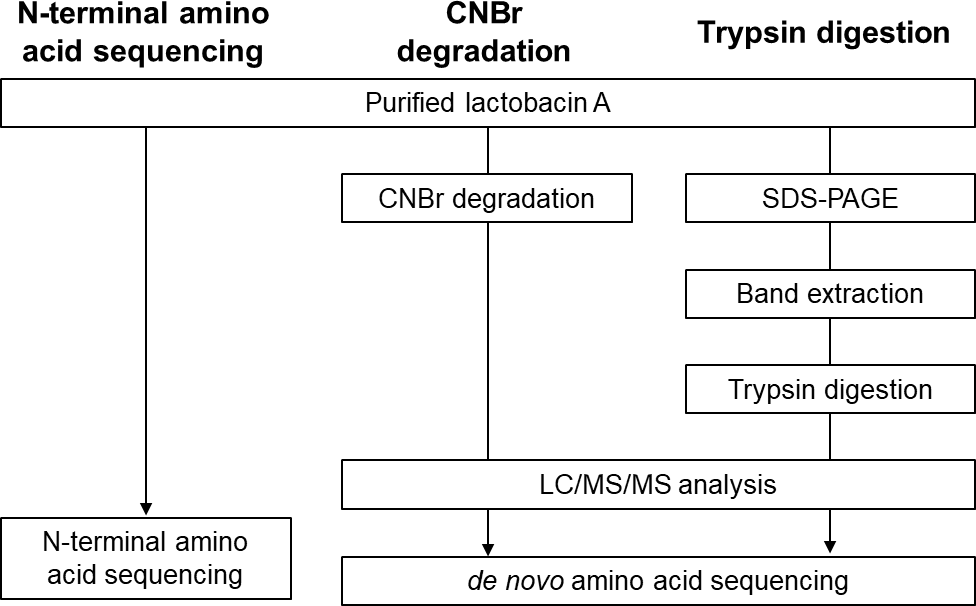
Workflow for analyzing the amino acid sequence of lactobacin A from *Liquorilactobacillus* sp*.* SC-2001.

The amino acid sequence of lactobacin A was determined using N-terminal sequencing and nano liquid chromatography-tandem mass spectrometry (nanoLC/MS/MS) *de novo* sequencing (Fig. 2). We first obtained the N-terminal amino acid sequence from the first to the 20th residue, excluding the fourth residue (Table 2). To identify the amino acid sequence of other parts of the peptide, we used various fragmentation methods and obtained sequence information by LC/MS/MS *de novo* sequencing (22). Treatment with cyanogen bromide (CNBr) (23), which cleaves the C-terminus of methionine, yielded two strong precursor ions, *m/z* 628.54 (*Z* = 5) and *m/z* 648.65 (*Z* = 3). The combined masses of the two fragments match the full-length peptide, accounting for a single modification during CNBr cleavage. This consistency indicates that the two fragments originate from the bacteriocin. By using PEAKS software, sequence reliability is indicated by the level of average local confidence (ALC); for the fragment at *m/z* 648.65, it was 99% ALC (Table 2). Since this fragment sequence did not match the N-terminal amino acid sequence, we considered it to be a C-terminal fragment sequence. On the other hand, the fragment at *m/z* 628.54 did not yield any promising candidate sequence with 90% or higher ALC.

**TABLE 2:** Amino acid sequences analyzed by fragmentation treatment of bacteriocin from *Liquorilactobacillus* sp. SC-2001

	Amino acid sequence	
N-terminal amino acid sequencing		
	KIIXISKWSYYNTKTKKYYA	

Treatment	Amino acid sequence	*de novo* ALC (%)
*de novo* amino acid sequencing		
CNBr degradation	STLGHTVVNGWVEHFPY	99
Trypsin digestion	KIIRSIKWSYYNTKTKKYYAD	96
	WSYYNTK	99
	YYADNSAIMSTLGHTVVNGWVEHFPY	>90

Next, to obtain further clues, trypsin digestion and sequence prediction were attempted. The fragment containing the sequence obtained by N-terminal amino acid sequencing was KIIRSLKWSYYNTKTKKYYYYAD with 96% ALC. From this result, the fourth residue of the bacteriocin was predicted to be R. Furthermore, the sequence WSYYNTK was also identified with 99% ALC. Based on the estimated molecular weight of the full-length sequence of lactobacin A, these fragments were still insufficient. Therefore, we looked for clues about the middle portion of the lactobacin A sequence. The fragment ions at *m/z* 991.13 yielded several candidate sequences with ALC scores above 90%, which were manually combined, suggesting that it was the middle to C-terminal portion of lactobacin A, namely YYADNSAIMSTLGHTVNGWVEHFPY. Together with the fragment sequence information, we deduced that the sequence of lactobacin A is KIIRISKWSYYNTKTKKYYADNSAIMSTLGHTVVNGWVEHFPY.

### Genomic and similarity analysis of *Liquorilactobacillus* sp*.* SC-2001

**Fig 3:**
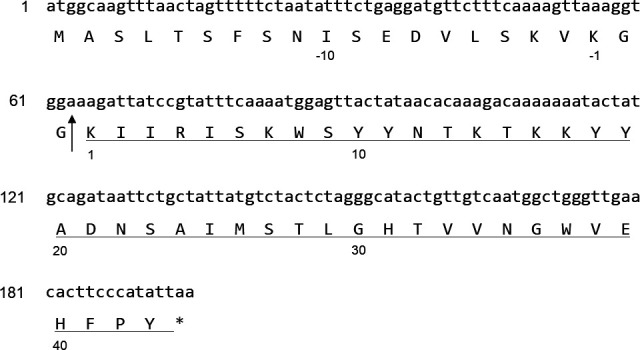
Nucleotide and deduced amino acid sequences of the *ORFX*. The vertical arrow indicates the site where the propeptide is cleaved to form lactobacin A. The asterisk identifies the translation stop codon. The underlined peptide sequence is in perfect agreement with the sequences deduced by structural analysis.

The genome of *Liquorilactobacillus* sp*.* SC-2001 was analyzed as described in the Materials and Methods section. To identify the gene encoding lactobacin A, DFAST was utilized for gene annotation, and an open reading frame (ORF) encoding an amino acid sequence identical to lactobacin A was found (24). Furthermore, a GG repeat, which is a conserved leader peptide cleavage site characteristic of Class II bacteriocins, was identified (25). As illustrated in Fig. 3, the predicted sequence of lactobacin A corresponds to the cleavage site after the GG motif and shows 100% identity to the mature sequence of *ORFX*. Lactobacin A shows no activity against *Listeria*, lacks cysteine residues and the “YGNGVXC” motif, and is a single, linear peptide that includes a leader peptide sequence. Therefore, it can be classified as Class IId.

**Fig 4:**
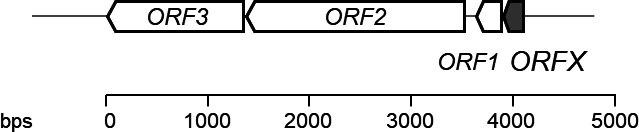
Genetic organization of the ORFX and its neighbors from *Liquorilactobacillus* sp*.* SC-2001.

In the genomic region surrounding *ORFX*, two adjacent ORFs, designated as *ORF2* and *ORF3*, were identified. The translated sequences of these ORFs showed homology to ATP-binding cassette (ABC) transporters (Fig. 4; Table 3). It is well-established that genes associated with antimicrobial peptides are often located near genes encoding resistance proteins, modifying enzymes, and transporters (26). Based on the genetic information obtained for lactobacin A, its sequence strongly suggests that it functions as an antimicrobial peptide.

However, genomic analysis using antiSMASH did not identify the bacteriocin gene as part of a bacteriocin cluster, implying that lactobacin A exhibits low similarity to previously reported bacteriocins.

**TABLE 3:** Neighboring *ORFX* genes and the structural gene for the bacteriocin from *Liquorilactobacillus* sp*.* SC-2001^*a*^

Gene	Amino acids (length)	Homolog	Protein identity (%)
*ORF3*	458	Bacteriocin ABC transporter of *L. plantarum* WCFS1	52
*ORF2*	717	Bacteriocin cleavage/export ABC transporter of *L. plantarum* WCFS1	69
*ORF1*	86	Protein with hypothetical function	

^*a*^ Protein identity is based on the amino acid sequences encoded by the corresponding genes in the gene clusters. Hypothetical function refers to the antiSMASH prediction (27). *orf*, open reading frame; ABC, ATP-binding cassette.

**Fig 5:**
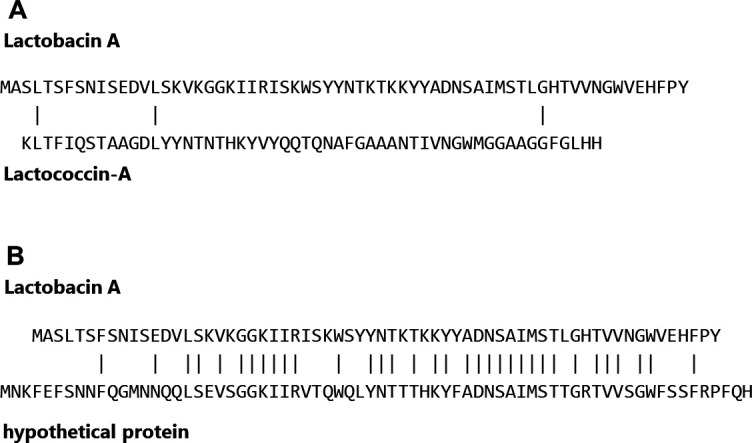
The results of the similarity search between the amino acid sequence of lactobacin A and the amino acid sequence of (**A**) lactococcin A and (**B**) hypothetical protein. BLAST was used for similarity search, amino acid sequences of polypeptides, including the leader sequence, were used for query, and “All non-redundant GenBank CDS translations + PDB + SwissProt + PIR + PRF excluding environmental samples from WGS projects” was used for the database.

To evaluate the novelty of this peptide sequence, we conducted a similarity search using known antimicrobial peptide sequences from the antimicrobial peptide databases DRAMP (28) and BACTIBASE (29, 30). The peptide most similar to this sequence in both databases was lactococcin-A; however, the match rate was only 5%. Additionally, a BLAST search of the peptide sequence identified a putative protein from *Leuconostoc aquikimchii* (WP_367973078.1) as the closest match, with a similarity rate of 57% (Fig. 5). Based on these results, we concluded that lactobacin A is novel.

### Activity evaluation using synthetic peptides

**Fig 6:**
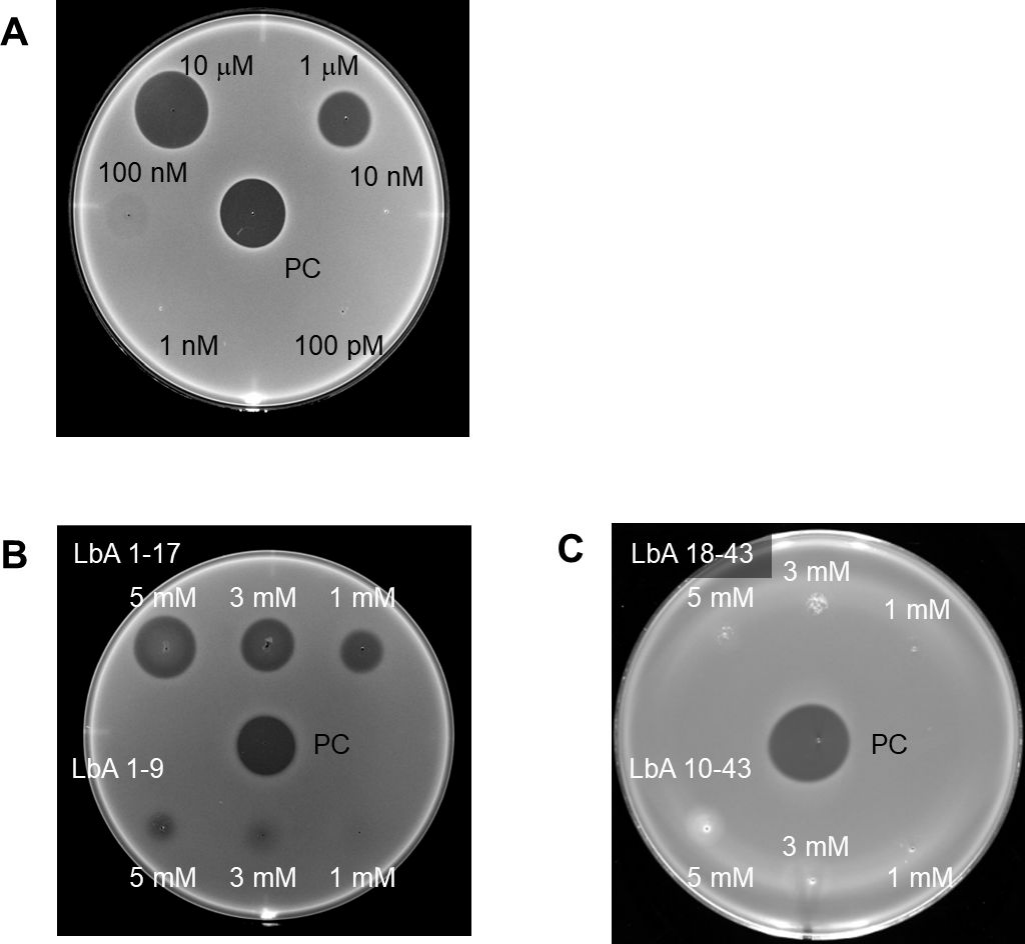
Antimicrobial activity of synthetic peptides derived from lactobacin A against *L. sakei*. The positive control (PC) was the supernatant from *Lactococcus lactis* ATCC 11,454, which produces nisin A. (**A**) The antimicrobial activity of full-length lactobacin A at various concentrations: 10 µM (top left), 1 µM (top right), 100 nM (middle left), 10 nM (middle right), 1 nM (bottom left), 100 pM (bottom right), and positive control (PC, center). (**B**) The antimicrobial activity of LbA 1–17 (top row: left, 5 mM; center, 3 mM; right, 1 mM), LbA 1–9 (bottom row: left, 5 mM; center, 3 mM; right, 1 mM), and positive control (PC, center). (**C**) The antimicrobial activity of LbA 18–43 (top row: left, 5 mM; center, 3 mM; right, 1 mM), LbA 10–43 (bottom row: left, 5 mM; center, 3 mM; right, 1 mM), and positive control (PC, center).

To confirm the antimicrobial activity of lactobacin A, the peptide was synthesized using solid-phase synthesis. nanoLC/MS/MS analysis of the synthetic lactobacin A produced a spectrum consistent with the results shown in Fig. 1. Synthetic lactobacin A demonstrated antimicrobial activity against *Lactilactobacillus sakei* at concentrations of 100 nM and above (Fig. 6A).

We evaluated the antimicrobial spectrum of both the culture supernatant and synthetic peptide A against the strains listed in Table 1. The culture supernatant exhibited inhibitory activity against several strains. In contrast, synthetic lactobacin A showed antimicrobial activity specific to *Streptococcus uberis* JCM 5709 (MIC 50 μM). These results suggest that the broader inhibitory spectrum observed with the culture supernatant may be due to the presence of other inhibitory substances in addition to lactobacin A. To identify the regions of lactobacin A responsible for antimicrobial activity, chemically synthesized partial peptides, LbA 1–17 and LbA 1–9, were evaluated (Table 4). At a concentration of 1 mM, LbA 1–17 produced a clear zone of growth inhibition, whereas LbA 1–9 exhibited only weak antimicrobial activity (Fig. 6B). In contrast, the C-terminal peptides corresponding to LbA 18–43 and LbA 10–43 showed no activity (data not shown).

**TABLE 4:** Synthetic peptides based on the amino acid sequence of lactobacin A

Name	Position	Sequence
Full length	1–43 aa	KIIRISKWSYYNTKTKKYYADNSAIMSTLGHTVVNGWVEHFPY
LbA 1–17	1–17 aa	KIIRISKWSYYNTKTKK
LbA 1–9	1–9 aa	KIIRISKWS
LbA 18–43	18–43 aa	YYADNSAIMSTLGHTVVNGWVEHFPY
LbA 10–43	10–43 aa	YYNTKTKKYYADNSAIMSTLGHTVVNGWVEHFPY

**Fig 7:**
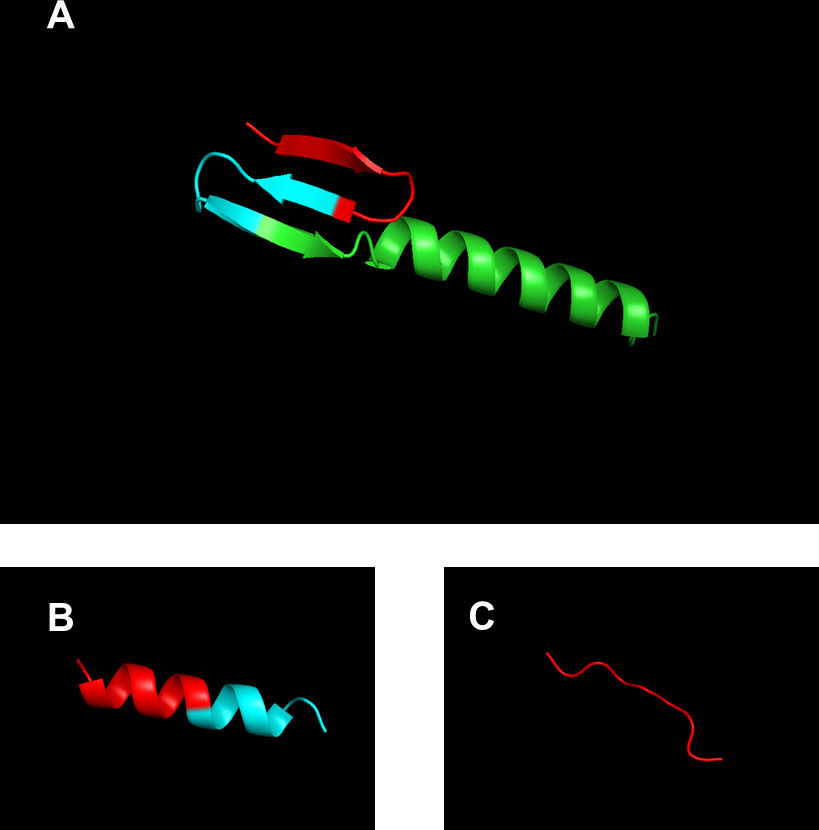
The bacteriocin structures were predicted by AlphaFold2. 1–9 aa is shown in red, 10–17 aa in cyan, and 18–43 aa in green. (**A**) Full-length lactobacin A. (**B**) LbA 1–17. (**C**) LbA 1–9.

Structural predictions for these peptides were performed using AlphaFold2 (Fig. 7). Full-length lactobacin A was predicted to adopt a helical structure at its C-terminal region, while LbA 1–17 was predicted to form a helical structure throughout its sequence. These structural features suggest that both full-length lactobacin A and LbA 1–17 may exert their antimicrobial effects by interacting with the target membrane. Many highly active peptides are reported to adopt an α-helical structure, and this conformation is known to facilitate efficient mechanisms such as membrane insertion, pore formation, and self-association (31, 32). In contrast, LbA 1–9 lacks a helical structure and contains numerous electropositively charged arginine and lysine residues. Indeed, previous studies have reported that non-helical antimicrobial peptides can induce extensive membrane disruption through the carpet model. Therefore, LbA 1–9 might also act via a similar mechanism (32, 33). Taken together, the findings indicate that helical structure may be associated with stronger antimicrobial activity, as observed for full-length lactobacin A and LbA 1–17, in contrast to LbA 1–9, which lacks this structural feature. Notably, the mode of action may shift depending on the truncation of the peptide, and further studies are required to elucidate the precise mechanisms underlying these observations.

### Stability of synthetic lactobacin A and its partial peptides under high pH, heat, and oxidative conditions

The stability of synthetic lactobacin A and its partial peptides was evaluated under various stress conditions, including high pH, heat treatment, and exposure to oxidation, and compared to that of nisin A.

The antimicrobial activity of lactobacin A and its partial peptides remained unaffected when exposed to high pH conditions (Table 5). Similarly, lactobacin A retained its activity even at elevated temperatures of 80°C or higher (Table 6). Furthermore, lactobacin A and its partial peptides demonstrated resistance to oxidative stress induced by hydrogen peroxide (Table 7). In comparison to nisin A, lactobacin A and its partial peptides exhibited superior stability under neutral to alkaline pH, high temperatures, and oxidative environments. These findings highlight the robustness of lactobacin A, making it a promising candidate for applications requiring stability under diverse and challenging conditions.

**TABLE 5:** Stability of synthetic lactobacin A peptides and purified nisin A subjected to different pH conditions for 24 h

	pH 4.0	pH 7.4	pH 10.0
Lactobacin A full length (10 μM)^*a*^	+++	+++	+++
LbA 1–17 (1 mM)^*a*^	++	++	++
LbA 1–9 (5 mM)^*a*^	+	+	+
Nisin A (10 μM)^*a*^	++	−	−

^*a*^ −, no antibacterial activity; +, antibacterial activity (inhibition zone diameter < 10 mm); ++, strong antibacterial activity (inhibition zone diameter 10–15 mm); +++, very strong antibacterial activity (inhibition zone diameter > 15 mm). Preparations of lactobacin A, its partial peptides, and nisin A at the indicated pH values were incubated. The residual activity was assayed using the *L. sakei* (NBRC15893)^T^ indicator strain. Samples dissolved in 0.1% TFA and not incubated were used as a control. It was confirmed that these control samples exhibited antimicrobial activity.

**TABLE 6:** Stability of synthetic lactobacin A and purified nisin A subjected to heat treatment at different temperatures

	80°C	100°C	121°C	Control
Lactobacin A full length (10 μM)^*a*^	+++	+++	++	+++
LbA 1–17 (1 mM)^*a*^	++	++	++	++
LbA 1–9 (5 mM)^*a*^	+	+	+	+
Nisin A (10 μM)^*a*^	−	−	−	++

^*a*^ −, no antibacterial activity; +, antibacterial activity (inhibition zone diameter < 10 mm); ++, strong antibacterial activity (inhibition zone diameter 10–15 mm); +++, very strong antibacterial activity (inhibition zone diameter > 15 mm). Lactobacin A, its partial peptides, and nisin A were heated at 80°C, 100°C, and 121°C. The residual activity was assayed using *L. sakei* (NBRC15893)^T^ as the indicator strain.

**TABLE 7:** Stability of synthetic lactobacin A and purified nisin A subjected to oxidation by hydrogen peroxide

	Oxidation	Control
Lactobacin A full length (10 μM)^*a*^	+	+
Nisin A (10 μM)^*a*^	−	+

^*a*^ −, no antibacterial activity; +, antibacterial activity (inhibition zone diameter > 13 mm). Lactobacin A and nisin A were treated with 10 mM hydrogen peroxide at 40°C for 6 h. The residual activity was assayed using *L. sakei* (NBRC15893)^T^ as the indicator strain.

### Conclusion

We successfully identified and characterized lactobacin A, a novel Class IId bacteriocin from *Liquorilactobacillus* sp*.* SC-2001. This strain is closely related to *L. mali* and represents the first reported case of a bacteriocin-producing strain within this lineage. Lactobacin A is a unique single-peptide bacteriocin, exhibiting low similarity to previously known bacteriocins. Genomic analysis revealed the presence of adjacent genes potentially involved in peptide transport and self-immunity mechanisms. Antibacterial assays demonstrated that the N-terminal sequence of lactobacin A plays a crucial role in its activity. Lactobacin A exhibited remarkable stability across a wide range of pH levels, elevated temperatures, and oxidative conditions. These characteristics position lactobacin A as a promising candidate for the development of novel antimicrobial agents.

## MATERIALS AND METHODS

### Bacterial strains and media

*Liquorilactobacillus* sp*.* SC-2001, a bacteriocin-producing strain, was isolated from *Quercus serrata* and identified by 16S rRNA gene sequencing as described in a previously published study (34). To precisely identify *Liquorilactobacillus* sp*.* SC-2001, the sugar fermentation pattern was assessed by using an API 50 CHL system (bioMérieux, Marcy l’Étoile, France), and analyzed with APILAB Plus software (bioMérieux). *Liquorilactobacillus* sp*.* SC-2001 was stored at –80°C in a stock solution containing 80% glycerol. Before use, *Liquorilactobacillus* sp*.* SC-2001 was propagated in de Man, Rogosa, and Sharpe (MRS) medium (Difco Laboratories, Detroit, MI, USA) at 30°C for 24 h.

### Antibacterial activity assay of culture supernatant of *Liquorilactobacillus* sp. SC-2001

Antibacterial activity of culture supernatant of *Liquorilactobacillus* sp. SC-2001 was tested on indicator strains (Table 1) and determined by a microdilution method. Briefly, 90 μL of the indicator strain culture (1–2 × 10^5^ CFU/mL) and 10 μL of twofold serial dilutions of culture supernatant of *Liquorilactobacillus* sp. SC-2001 were added to 96-well plates. The plates were incubated at 35°C until the bacteria were fully grown, and the absorbances of the mixed solutions were assayed using a microplate reader at 600 nm. The minimum inhibitory concentration was defined as the lowest growth-inhibitory concentration.

### Bacteriocin activity test

To evaluate the antimicrobial activity of the bacteriocins, the spot-on-lawn method was used as previously described (35–37). Unless otherwise stated, *L. sakei* (NBRC15893^T^) was used as an indicator strain. In brief, 10 μL of the test solution was spotted on a double-layered agar plate. The upper layer consisted of *Lactobacilli* agar AOAC (Difco Laboratories) incubated with an overnight culture of the indicator strain at a density of 4 × 10^7^ CFU/mL. The lower layer was made up of MRS medium containing 2% agar. Following the application of the test solution, the plates were incubated overnight at 30°C. The antimicrobial activity was evaluated based on the size of the clear zone.

### Sequence estimation of lactobacin A by genomic analysis

Genomic DNA of *Liquorilactobacillus* sp. SC-2001 was sequenced using both short-read (NovaSeq6000) and long-read (PacBio RS II) sequencing platforms. The sequencing reads were assembled with Unicycler (33), resulting in two cycler contigs corresponding to a chromosome and a plasmid. The total genome size was 2.4 Mbp. The genome was considered complete. The 16S rRNA gene sequence was extracted from the assembled genome and used for species identification. Open reading frame (ORF) and gene annotation were performed using DFAST (24). Homologous peptide sequences to known bacteriocins, as listed in BACTIBASE (29, 30), were searched using HMMER (38). Corresponding bacteriocin biosynthetic gene clusters were detected using antiSMASH (27).

### Bacteriocin purification

Bacteriocins were extracted from 1 L of culture supernatant, which was obtained by centrifugation at 6,000 × *g* for 10 min and filtered through a 0.22 mm cellulose acetate membrane to remove cells. Ammonium sulfate was added to 500 mL of cell-free culture supernatant to achieve 80% saturation, and the mixture was stirred overnight at 6°C to precipitate the proteins. The precipitate was collected by centrifugation at 13,000 × *g* for 60 min and redissolved in 50 mL of 50 mM sodium phosphate buffer (pH 5.6; Buffer A). The solution was loaded onto a Celite column (Biotage, Uppsala, Sweden) to remove impurities. Following this, a buffer exchange was performed using an Amicon Ultra 3 kDa membrane (Merck Millipore, Darmstadt, Germany) pre-equilibrated with Buffer A. The resulting solution was loaded onto a Hitrap SP FF 5-Ml column (GE Healthcare, Uppsala, Sweden). Elution was performed using a gradient of Buffer B (50 mM sodium phosphate buffer [pH 5.6] containing 1 M NaCl) in Buffer A at a flow rate of 1 mL/min. The gradient conditions were as follows: initial mobile phase 0% Buffer B, gradually increasing to 100% Buffer B over 25 min. Fractions were collected in 1 mL increments. For additional purification, the active fractions were pooled and applied to a 3 mL Resource RPC column (GE Healthcare). Elution was performed at a flow rate of 1 mL/min using a linear gradient of 0% to 100% of ethanol in 0.1% formic acid for 30 min, with fractions collected every 1 mL. The purified active fractions were stored at –30°C after solvent removal using a CentriVap Benchtop Vacuum Concentrator (Labconco, Kansas City, MO, USA). The concentration of the purified bacteriocin was determined using a Pierce BCA protein assay kit (Thermo Fisher Scientific Inc., Waltham, MA, USA). The antibacterial activity of the fractions obtained at each purification step was evaluated as described above.

### Mass spectrometry

The purified lactobacin A was measured using nano liquid chromatography-tandem mass spectrometry (LC/MS/MS) with an Ultimate 3000 RSLCnano system (Thermo Fisher Scientific Inc.) and Q Exactive HF system (Thermo Fisher Scientific Inc.). Separation of the bacteriocin was achieved on an Acclaim PepMap RSLC column (75 mm × 50 cm nanoViper C18, 2 mm, 100 Å; Thermo Fisher Scientific Inc.) (22). The analysis was conducted at a flow rate of 250 nL/min with the linear gradient of 5% to 55% of methanol in 0.5% formic acid for 90 min. The resulting fragmentation spectra were examined for antimicrobial peptide sequencing using PEAKS Studio version 10 (Bioinformatics Solutions Inc., Waterloo, ON, Canada).

### Tryptic digestion of purified lactobacin A

The purified lactobacin A was separated using Tris-Tricine sodium dodecyl sulfate polyacrylamide gel electrophoresis (SDS-PAGE). The specific bands were extracted with the Protein Extraction Kit from Gel Slices (Cosmo Bio, Tokyo, Japan). The proteins were digested using AccuMAP Low pH Protein Digestion Kits (Promega, Madison, WI, USA). For reductive alkylation, 25 µL of rLys-C was added and incubated at 37°C for 4 h, according to the manufacturer’s protocol. The mixture was diluted, and another 25 µL of rLys-C was added, followed by incubation at 37°C for 16 h. Subsequently, 20 µL of trypsin was added, and the mixture was incubated at 37°C for 3 h. The reaction was stopped by adding 20% trifluoroacetic acid (TFA). The digested samples were then desalted, and the reaction progress was monitored using liquid chromatography-mass spectroscopy (LC/MS).

### Cyanogen bromide treatment of purified lactobacin A

Purified lactobacin A was dissolved in 100 μL of 70% (vol/vol) formic acid containing 50 μmol of CNBr. The reaction was carried out at room temperature for 18 h as detailed in a previous report (23). The resulting degraded peptides were desalted on an SDB tip (GL Science, Tokyo, Japan) (39) and analyzed by LC/MS.

### Computer analysis of amino acid sequences

Database searches were performed using National Center for Biotechnology Information BLAST (http://www.ncbi.nlm.nih.gov/BLAST/).

### Lactobacin A and its partial sequence peptide synthesis

The chemically synthesized peptides used in this experiment were purchased from Hokkaido System Science (Hokkaido, Japan).

### Nisin A preparation

The method of nisin A enrichment was performed following a previous report: 1 g of nisin from *Lactococcus lactis* 2.5% (Sigma Aldrich, St. Louis, MO), containing approximately 25 mg of nisin A, was weighed, 25 mL of water was added, and the mixture was stirred at room temperature for 15 min. The precipitate was separated by centrifugation (15 min, 2,500 rpm), and only the supernatant was transferred to another tube. To this solution, 20 mL of dichloromethane was added. The upper and lower layers were removed to collect the white precipitate that formed between the aqueous and organic layers. Further centrifugation (15 min, 2,500 rpm) was performed to remove the upper and lower layers again. The desired intermediate layer was dried in a decompression centrifuge and redissolved in 7.45 mL of water to obtain a 1 mM nisin purified product. This purified product was used as a positive control for nisin A in each stability study.

### pH and thermal stability test of synthetic bacteriocins

To evaluate the effect of pH on bacteriocin activity, synthetic lactobacin A molecules (full length, and partial peptides 1–17th and 1–9th of lactobacin A) and purified nisin A were adjusted to a pH range of 4.0–10.0 with the following buffers (50 mM): sodium acetate-acetic acid buffer (pH 4.0), sodium phosphate (pH 7.4), and glycine-NaOH (pH 10.0). The samples were incubated at 30°C with shaking at 300 rpm. All experiments were performed in six biological replicates. To test thermal stability, the synthetic lactobacin A molecules and purified nisin A were heat-treated and assessed for their antimicrobial activity. The samples were heated at 80°C, 100°C, or 121°C. Bacteriocin activity was measured by the spot-on-lawn method as previously described. All experiments were performed in two biological replicates, except for those conducted at 121°C.

### Oxidant stability test of bacteriocins

Lactobacin A and nisin A were assessed for antimicrobial activity after oxidation treatment. The oxidation treatment was conducted as previously reported (40): hydrogen peroxide was added to a 1 mM solution of lactobacin A or nisin A to achieve a final concentration of 10 mM, and the mixture was allowed to react at 40°C for 6 h. Upon completion of the reaction, the solution was diluted with water to a concentration of 10 μM. The antimicrobial activity of the oxidized polypeptides was evaluated by the spot-on-lawn method, as previously described for antimicrobial activity testing. All experiments were performed in duplicate biological replicates.

## Data Availability

The nucleotide sequence determined in this study has been deposited in the DDBJ database under the accession no. LC817274.

## SUPPLEMENTAL MATERIAL

Figure S1 spectrum.01382-25-s0001.tif The bacteriocin structures were predicted by AlphaFold2.

Supplemental legend spectrum.01382-25-s0002.docx Fermentability test and Physiological and biochemical characterization test.

Supplemental table spectrum.01382-25-s0003.docx Tables S1 and S2.

ASM does not own the copyrights to Supplemental Material that may be linked to, or accessed through, an article. The authors have granted ASM a non-exclusive, world-wide license to publish the Supplemental Material files. Please contact the corresponding author directly for reuse.
