# Supplementary figures and images for: Structural and functional insights into lactobacin A: a novel non-pediocin-like bacteriocin from a Liquorilactobacillus strain related to L. mali
Source: Microbiol Spectr. 2026 Mar 9;14(4):e01382-25. doi: 10.1128/spectrum.01382-25 (PMC13055208; doi:10.1128/spectrum.01382-25)

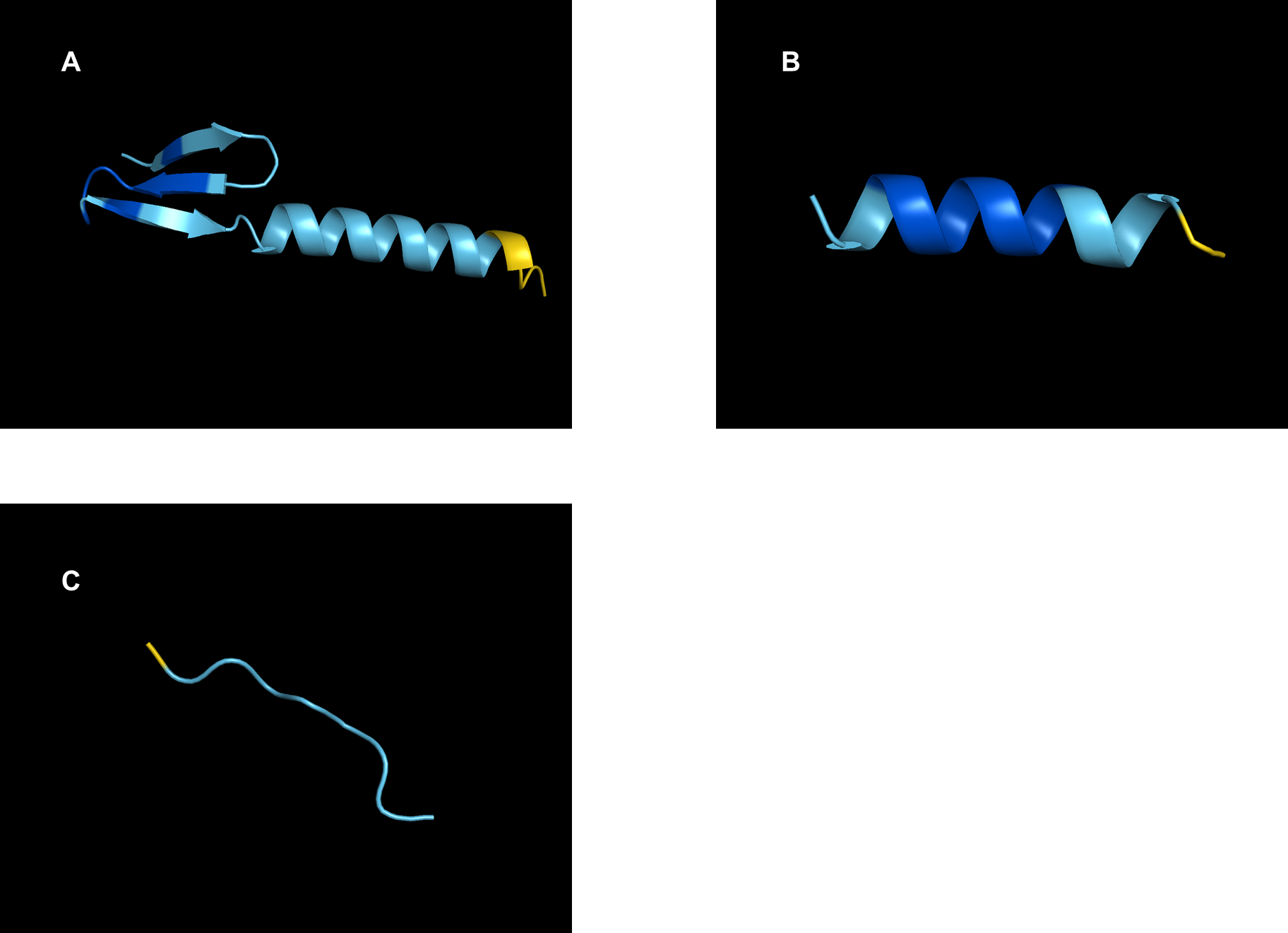

Supplement: Figure S1 — The bacteriocin structures were predicted by AlphaFold2. [file spectrum.01382-25-s0001.tif]
