# Supplementary material for: Structural and functional insights into lactobacin A: a novel non-pediocin-like bacteriocin from a Liquorilactobacillus strain related to L. mali
Source: Microbiol Spectr. 2026 Mar 9;14(4):e01382-25. doi: 10.1128/spectrum.01382-25 (PMC13055208; doi:10.1128/spectrum.01382-25)
Supplement: Supplemental legend — Fermentability test and Physiological and biochemical characterization test. [file spectrum.01382-25-s0002.docx]

**Fig. S1.** The bacteriocin structures were predicted by AlphaFold2. (A) full-length lactobacin A. (B) LbA 1-17. (C) LbA-1-9. The structures are colored according to the predicted Local Distance Difference Test (pLDDT) confidence scores: very high (pLDDT > 90, dark blue), confident (90 > pLDDT > 70, light blue), low (70 > pLDDT > 50, yellow), and very low (pLDDT < 50, orange).
