# Supplementary material for: Structural and functional insights into lactobacin A: a novel non-pediocin-like bacteriocin from a Liquorilactobacillus strain related to L. mali
Source: Microbiol Spectr. 2026 Mar 9;14(4):e01382-25. doi: 10.1128/spectrum.01382-25 (PMC13055208; doi:10.1128/spectrum.01382-25)
Supplement: Supplemental table — Tables S1 and S2. [file spectrum.01382-25-s0003.docx]

**Supplemental Materials**

**Table S1.** Fermentability test for *Liquorilactobacillus sp.* SC-2001

| Item | Substrate component | Test results | Item | Substrate component | Test results |
| --- | --- | --- | --- | --- | --- |
| 0 | Control | - | 25 | Esculin | + |
| 1 | Glycerol | - | 26 | Salicin | + |
| 2 | Erythritol | - | 27 | Cellobiose | + |
| 3 | D-Arabinose | - | 28 | Maltose | + |
| 4 | L-Arabinose | - | 29 | Lactose | + |
| 5 | Ribose | - | 30 | Melibiose | + |
| 6 | D-Xylose | - | 31 | Sucrose | + |
| 7 | L-Xylose | - | 32 | Trehalose | + |
| 8 | Adonitol | - | 33 | Inulin | - |
| 9 | β-Methyl-D-xylose | - | 34 | Melezitose | - |
| 10 | Galactose | - | 35 | Raffinose | - |
| 11 | Glucose | + | 36 | Starch | - |
| 12 | Fructose | + | 37 | Glycogen | - |
| 13 | Mannose | + | 38 | Xylitol | - |
| 14 | Sorbose | - | 39 | Gentiobiose | - |
| 15 | Rhamnose | - | 40 | D-Turanose | - |
| 16 | Dulcitol | - | 41 | D-Lyxose | - |
| 17 | Inositol | - | 42 | D-Tagatose | - |
| 18 | Mannitol | - | 43 | D-Fucose | - |
| 19 | Sorbitol | - | 44 | L-Fucose | - |
| 20 | α-Methyl-D-mannoside | - | 45 | D-Arabitol | - |
| 21 | α-Methyl-D-glucoside | - | 46 | L-Arabitol | - |
| 22 | N-Acetylglucosamine | + | 47 | Gluconate | - |
| 23 | Amygdalin | - | 48 | 2-Ketogluconate | - |
| 24 | Arbutin | - | 49 | 5-Ketogluconate | - |
| + : Positive, - : Negative | |  |  |  |  |

**Table S2.** Physiological and biochemical characterization test for *Liquorilactobacillus sp.* SC-2001

| Test item | |  |
| --- | --- | --- |
| Culture temperature | | 30°C |
| Cell morphology | | Rod (0.7-0.8×5.0-10.0 µm) |
| Gram stainability | | + |
| Presence or absence of spore | | - |
| Motility | | + |
| Colony morphology | Medium | MRS agar medium |
|  | Culture time | 48 hours |
|  | Diameter | 1 to 2 mm |
|  | Color Tones | White |
|  | Shape | Circular |
|  | Raised state | Lens shape |
|  | Circumferential edge | Entire edge |
|  | Surface shape | etc. |
|  | Transparency | Opaque |
|  | Consistency | Butter-like |
| Growth temperature test | 15°C | + |
|  | 37°C | + |
|  | 45°C | - |
| Arginine dihydrolase activity | | - |
| Catalase reaction | | - |
| Oxidase reaction | | - |
| Acid/gas production from glucose (Acid production/gas production) | | +/- |
| O/F test (Oxidation/Fermentation) | | +/+ |
| + : Positive, - : Negative |  |  |
